# Supplementary material for: Soil Lead (Pb) in New Orleans: A Spatiotemporal and Racial Analysis
Source: Int J Environ Res Public Health. 2021 Feb 1;18(3):1314. doi: 10.3390/ijerph18031314 (PMC7908533; doi:10.3390/ijerph18031314)
Supplement: Supplementary file 1 [file ijerph-18-01314-s001.zip › supple s1.docx]

**Table S1.** Kendall’s tau-b correlation results and P-values. The potential for Pb exposure and comorbidity is highest among the people living within the inner city of New Orleans.
